# Assessment of an automated approach for variant interpretation in screening for monogenic disorders: A single‐center study

**DOI:** 10.1002/mgg3.2085

**Published:** 2022-11-05

**Authors:** Bryan J. Gall, Trevor B. Smart, Robin Munch, Supraja Kolluri, Hamsa Tadepally, Karen Phaik Har Lim, Zachary P. Demko, Peter Benn, Vivienne Souter, Nina Sanapareddy, Dianne Keen‐Kim

**Affiliations:** ^1^ Natera Inc. Austin Texas USA; ^2^ Genetics and Genome Sciences University of Connecticut Health Center Farmington Connecticut USA

**Keywords:** automated variant classification, genetic testing, manual curation, monogenic disorder, variant classification, variant interpretation, variants of uncertain significance

## Abstract

**Background:**

Automation has been introduced into variant interpretation, but it is not known how automated variant interpretation performs on a stand‐alone basis. The purpose of this study was to evaluate a fully automated computerized approach.

**Method:**

We reviewed all variants encountered in a set of carrier screening panels over a 1‐year interval. Observed variants with high‐confidence ClinVar interpretations were included in the analysis; those without high‐confidence ClinVar entries were excluded.

**Results:**

Discrepancy rates between automated interpretations and high‐confidence ClinVar entries were analyzed. Of the variants interpreted as positive (likely pathogenic or pathogenic) based on ClinVar information, 22.6% were classified as negative (variants of uncertain significance, likely benign or benign) variants by the automated method. Of the ClinVar negative variants, 1.7% were classified as positive by the automated software. On a per‐case basis, which accounts for variant frequency, 63.4% of cases with a ClinVar high‐confidence positive variant were classified as negative by the automated method.

**Conclusion:**

While automation in genetic variant interpretation holds promise, there is still a need for manual review of the output. Additional validation of automated variant interpretation methods should be conducted.

## Graphical abstract

Here, we performed a comparative analysis of a fully automated curation of variant classification versus a process that included an additional manual component. The comparison was carried out for a set of variants where there was high confidence in their pathogenic classification, based on ClinVar entries. We found that a high proportion of automated interpretations (22.6% of positive and 1.7% of negative variants) were reclassified when there was a manual review. We conclude that manual review of the output from the automated variant classifier is currently essential.

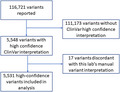

## 1 INTRODUCTION

Genetic variant interpretation is a critical part of clinical genetic testing, consisting of the review of literature, databases, and predictive algorithms to convert genomic data into actionable clinical results following the American College of Medical Genetics and Genomics (ACMG) and the Association for Molecular Pathology (AMP) published guidelines (Richards et al., 2015). The goal is to provide clinicians with clinical assessments of each variant that will facilitate shared decision making between patients and their test providers. Despite these guidelines, there are many nuances in variant interpretation that require significant input from trained scientists who are familiar with variant interpretation (Strande et al., 2018). One of the resources created by the National Institutes of Health to potentially minimize variability in variant interpretation across multiple labs is ClinVar. ClinVar is a public, open‐access archive of human genetic variants with their pathogenicity based on submissions from multiple commercial and academic genetic clinical laboratories (Landrum et al., 2018). As of May 2022, there were over 2000 laboratories, including Natera, submitting variant interpretations to ClinVar. Although submissions for many variants may have conflicting interpretations (Amendola et al., 2016; Harrison et al., 2018; Landrum & Kattman, 2018; Vail et al., 2015; Van Driest et al., 2016), variants with concordant entries from multiple submitters with assertion criteria consistent with ACMG/AMP guidelines can be used as a reference dataset to assess alternative approaches to variant interpretation.

Early in the clinical adoption of next‐generation sequencing (NGS), allowing broad panel genetic testing, significant manual effort was required to assess each genetic variant. Manual review is extremely labor intensive, requiring large teams of highly trained variant scientists to collect literature from diverse databases and other resources. This literature must then be critically evaluated and integrated with additional data from population frequency databases (e.g., gnomAD [Karczewski et al., 2020]), historical interpretation databases (e.g., ClinVar [Landrum et al., 2018]), and in silico functional prediction tools (e.g., Mutation Taster [Schwarz et al., 2010] and CADD [Kircher et al., 2014]). The challenge of scaling up variant interpretation may have hampered the early growth of genetic testing and has led to the development of automated software, like Qiagen Clinical Insights (QCI), which accelerates data collection by up to 80% (Cox et al., 2016). The need for large‐scale clinical genomics interpretation has further driven the development of completely automated variant interpretation software that streamlines the process of variant interpretation. Automated variant interpretation software is potentially well‐equipped to draw on standardized resources, such as population frequency databases, historical interpretation databases, and in silico functional prediction tools. However, automated methods may miss more complex clinical information from literature that needs expert interpretation, which is why many major clinical genetics companies continue to perform a manual review, using automated software only as a part of data collection. More recently, in order to address the increasing demand for variant interpretation, automated software has been created with the hope that this approach could accurately classify variants in a stand‐alone manner thus meeting the rapidly increasing demand for variant interpretation (Chunn et al., 2020; Furness, 2016; Perakis et al., 2020; Ravichandran et al., 2019).

Although there are some preliminary publications on automated variant curation software (Chunn et al., 2020; Furness, 2016; Perakis et al., 2020; Ravichandran et al., 2019), there is minimal peer‐reviewed evidence from independent laboratories, focusing on clinical cohorts or providing data evaluating concordance between fully automated and manual assessment of variants. This study was a large retrospective analysis of carrier‐screening data designed to assess the variant interpretation of an automated method by measuring concordance rates between its interpretations and those of the consensus of manual curations as found in the ClinVar database.

## 2 MATERIALS AND METHODS

This retrospective cohort study included individuals who received results for the Natera Horizon™ carrier screen between November 4, 2019, and November 4, 2020, and who consented to additional research studies. This testing included panels of up to 274 genes with variants associated with clinically significant disorders. Details of the populations receiving this testing have been described elsewhere (Westemeyer et al., 2020). Data were de‐identified, and an IRB exemption was obtained for publication of this analysis [E&I Review Services, Corte Madera, CA; IRB number: 17113‐05].

In our laboratory, variant interpretation scientists manually review the raw data output from an automated variant classifier, along with many other sources of information, prior to generation of a clinical report. This process is referred to as “manual interpretation”. Determinations based on computer interpretation alone, are referred to as “automated interpretations”. Automated interpretation involves the evaluation of variants identified by the carrier screening panel using Qiagen Clinical Insights (QCI) Clinical Decision Support Software (Qiagen Inc., Redwood City, CA). QCI employs proprietary automated methods to identify literature and invoke ACMG/AMP criteria. While the processes involved in QCI's software are not fully specified, it apparently collects data from publicly available sources (e.g., ClinVar, the Human Gene Mutation Database [HGMD], gnomAD, CADD, PubMed) to evaluate if each of the 27 ACMG/AMP criteria can be applied to the variant of interest (Richards et al., 2015). QCI then uses the approach recommended by Richards et al. (2015) to determine the final interpretation of the variant.

“Manual interpretation” involved a search of Leiden Open Variation Database (LOVD), the Human HGMD, ClinVar, Google Scholar, and other gene‐specific databases, as available. Further review of the criteria invoked by the automated software was performed to evaluate accuracy. An interpretation was ultimately made by a trained variant curator by considering all the evidence gathered during the various searches. For this study, “automated interpretation” refers to the use of the software's predicted interpretation without manual input from the end user (e.g., laboratory staff).

Variant reporting followed ACMG/AMP guidelines with variants considered P (pathogenic) and LP (likely pathogenic) reported as ‘positive’, and B (benign), LB (likely benign), and VUS (variants of uncertain significance) classified as ‘negative’. Although the ACMG/AMP guidelines do not explicitly consider positive and negative variants, we used this grouping as part of our carrier‐screening reporting. In this setting, LP and P variants have sufficient evidence to justify clinical actionability (i.e., testing a partner, counseling regarding risk, etc.), while VUS, LB, and B variants are considered negative because carrier patients are assumed to be healthy, and there is insufficient evidence to justify further testing or altering risk (Fridman et al., 2020; Silver & Norton, 2021). Reports for positive cases contained a description of the variant using recommended nomenclature, interpretative comments, and recommendations for genetic counseling.

Consistent with industry standards, all variant interpretations performed by our laboratory were considered to be current for a six‐month period, and during that period all new individuals with the same variant were automatically reported based on the previous interpretation by our bioinformatic pipeline. This process automates a portion of the variant interpretation process, while maintaining a rigorous standard of manual review. All variants identified without a full review within the prior 6 months are subject to manual review for new evidence that could affect the final interpretation.

We considered a ClinVar entry as “high confidence” if the entry for a variant contained two or more documented submissions of the variant, and those submissions had concordant assessments with respect to their stratification into the ‘positive’ and ‘negative’ groupings. The ClinVar “high confidence” set further required submissions to come from a subset of commercial laboratories in the United States that provided supporting information consistent with ACMG/AMP guidelines for the majority of their ClinVar submissions. Where laboratories disagreed on the sub‐type of interpretation but were both in agreement on positive or negative (e.g., one laboratory reported LP and another reported P), the variant was included in the analysis. The ClinVar dataset was based on all entries prior to November 4, 2020. Variants for which there was insufficient ClinVar data, discordance among ClinVar submitter assessments, or ClinVar interpretations conflicted with this laboratory's ACMG/AMP‐consistent manual interpretation, were excluded. The remaining variants provided the basis for the evaluation of the automated interpretations.

We categorized the reasons for discordance between the consensus manual and automated interpretation approaches. The categories of discrepancy include:

1. “Case criteria not invoked by automation” represents variant cases where the automated method did not apply ACMG/AMP criteria based on publications that were considered relevant by manual review. This discordance was based on the classifications of pathogenic when in *trans* with a pathogenic variant (PM3), known co‐segregation with disease (PP1), observed in healthy individuals (BS2), and observed in *trans* or *cis* with a known pathogenic variant (BP2) with these codes fully defined by Richards et al., 2015.
2. “High‐frequency pathogenic variant” includes variants classified as negative by automation due to higher than expected allele frequency, but where high frequency could be attributable to a sub‐population (e.g., Ashkenazi Jewish), and manual review considered the clinical evidence sufficient to support pathogenicity. This discordance arises when the allele frequency is greater than that expected for the disorder (BS1) The consequence of invoking this criterion is that the variant is classified as VUS by automated interpretation software but LP/P by manual interpretation.
3. “Atypical, subclinical, or somatic phenotype” includes variants where the automated process invokes ACMG/AMP criteria based on a phenotype that is inconsistent with that expected (e.g., cancer for a *CFTR* variant [NM_000492.4]). This discordance results from invoking PM3, PP1, BS2, and BP2 (Richards et al., 2015). In this scenario, the phenotype is likely a result of variants in other genes.
4. “Functional criteria not invoked by automation” represents situations where functional data (e.g., enzyme activity, transcript splicing, etc.) is used to invoke ACMG/AMP criteria by manual review, but not by the automated method. This discordance arises when there are accepted functional studies indicating a deleterious effect (PS3) or there is no deleterious effect (BS3) (Richards et al., 2015).
5. “Functional prediction discordance” represents situations where the automated process used in silico data to invoke ACMG/AMP protein functional prediction criteria based on a few primary in silico tools (i.e., CADD (Kircher et al., 2014), MaxEntScan (Yeo & Burge, 2004)), but manual review reached different conclusions based on the consideration of evidence from additional in silico tools (i.e., PolyPhen (Sunyaev et al., 2001), SIFT (Ng & Henikoff, 2001), Mutation Taster (Schwarz et al., 2010), GeneSplicer (Pertea et al., 2001)). In silico tools are computational assessments of the predicted effect of variants on a protein or transcript using a variety of data including, but not limited to, conservation across species, functional domains, predicted change in protein stability based on amino acid chemical properties, and calculated risk of creating or destroying splicing elements. These programs do not consider clinical evidence or functional studies. For nonsynonymous variants, manual review invoked in silico ACMG/AMP criteria based on the predicted effect of variants from a majority of prediction tools (e.g., CADD, PolyPhen, SIFT, and Mutation Taster) or if both commonly used splicing tools were concordant for synonymous or intronic variants (e.g., MaxEntScan and GeneSplicer). Discordance in ACMG/AMP criteria for predicted functional domains is also included, when the automated method considered a variant to be in a predicted functional domain or hotspot, but manual review could not confirm in UniProt (UniProt, 2021). This discordance arises when considering mutation hot spots or critical domains (PM1), or other computational evidence for a deleterious (PP3) or non‐deleterious effect (BP4) (Richards et al., 2015).
6. “Variable penetrance” represents variants where healthy individuals are listed in a population database (e.g., gnomAD) or in the literature, but manual review of the clinical, functional, other databases, and predictive evidence supported the pathogenicity of the variant (BS2). This makes the variant VUS by automated interpretation software and LP/P by manual interpretation.
7. “Expert decision” describes variants where there was ambiguity in the evidence. These variants required evaluation from board‐certified clinical experts to make a subjective interpretation for the variant and where this expert assessment was in agreement with the consensus of manual interpretations in ClinVar, but not with the automated interpretation. This discordance can be the result of ambiguity of any ACMG/AMP criteria, but the conclusions are in agreement with subjective interpretation from multiple clinical laboratories.
8. “ClinVar entry not identified by automated program” represents variants where the automated process did not identify a ClinVar submission that was used to invoke ACMG/AMP criteria during manual review. This discordance is based on evidence of pathogenicity (PP5) or a benign effect (BP6), but the information was unavailable to the laboratory (Richards et al., 2015).
9. “Late null variant” represents a null variant (e.g., frameshift, nonsense, canonical splice site variant) classified as positive by the automated method that occurs in the last exon or in the last 50 base pairs of the penultimate exon (i.e., such that nonsense‐mediated decay would not be predicted (Popp & Maquat, 2013)). Additional evidence was insufficient to support the pathogenicity of the variant as nonsense‐mediated decay of the transcript. Our laboratory makes exceptions for these PVS1 variants that are 3′ to the nonsense‐mediated decay region, when there are clinical cases with LP/P variants 3′ to the variant of interest, as recommended by ClinGen updates to the ACMG/AMP guidelines (Abou Tayoun et al., 2018). The discordance arises when the automated software invokes PVS1 while manual review concluded it was not applicable.

We then evaluated the performance of the automated interpretations, relative to the consensus manual interpretations, in two ways, first on a per‐variant basis, and second, on a per‐case basis. The latter approach accounts for the observed variant frequency in the study cohort.

## 3 RESULTS

**FIGURE 1:**
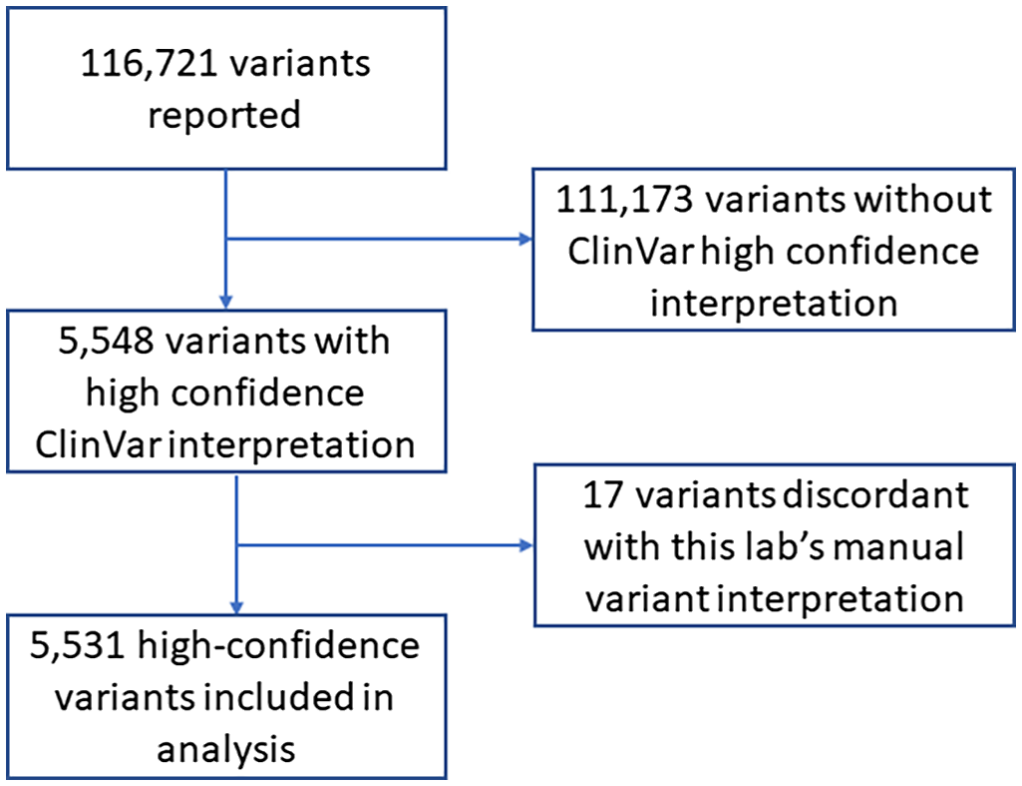
CONSORT diagram for variant interpretations that were considered as high‐confidence entries for this study

During the one‐year study interval, there were 116,721 unique variant interpretations, of which 5,548 different variants were considered high confidence based on analysis of ClinVar entries. Of these, 0.3% (17/5,548) ClinVar interpretations were discrepant with the manual interpretation made by our laboratory (Figure 1). These 17 cases were excluded from the study.   Table S1 provides details of these 17 discrepancies and why our laboratory did not agree with ClinVar interpretation. The remaining 5,531 variants were used as consensus ClinVar high‐confidence interpretations in the analysis. Of these, 23.5% (1,299/5,531) were classified as positive and 76.5% (4,232/5,531) were classified as negative.

Table 1 summarizes the concordance rates between the automated variant interpretation and the high‐confidence manual variant interpretations on a per‐variant basis. Of the 1,299 positive variants interpreted by the manual procedure, 1,004 (77.3%) were also reported as positive by the automated approach. For the 4,232 variants that were assigned as negative based on the manual approach, 4,159 (98.3%) were also computed as negative when using the automated approach. Overall, for all 5,531 variants, the interpretation of 5,163 (93.3%) was concordant between the automated and consensus manual curation process.

**TABLE 1:** Comparison of the fully automated variant interpretation methodology to the high‐confidence manual variant interpretations

	High‐confidence manually interpreted variants			
		Positive (%)	Negative (%)	Total
Fully Automated Variant calls	Positive	1,004 (77.3)	73 (1.7)	
	Negative	295 (22.7)	4,159 (98.3)	
	Total	1,299	4232	**5,531**

Table 2 summarizes the reasons for discordance between the manual high confidence and automated approaches. The most common reason for discordancy (46.5% of cases; *n* = 167) was the automated method not applying ACMG/AMP case criteria from publications considered relevant by manual review. In this category, the automated program identified the literature within the variant bibliography in 166 of 167 (99.4%) cases, but manual review was required to fully extract the relevant information from the literature. Another significant cause for discordancy was high‐frequency pathogenic variants (15.9% of cases; *n* = 57), which represented variants with clinical evidence considered to be significant by manual review to support their pathogenicity but with higher than was expected allele frequency attributable to a sub‐population (e.g., Ashkenazi Jewish).

**TABLE 2:** Reasons for discordance between fully automated and manual high‐confidence variant classifications

Reason for discordancy	Variant counts	Percent
Case criteria not invoked by automation	167	46.5%
High‐frequency pathogenic variant	57	15.9%
Functional criteria not invoked by automation	37	10.3%
Atypical, subclinical, or somatic phenotype	35	9.7%
Expert decision	24	6.7%
Functional prediction discordance	21	5.8%
Variable penetrance	12	3.3%
ClinVar entry not identified by automated program	5	1.4%
Late null variant	1	0.3%
Total	359	100.0%

The per‐case rates of concordance, which account for variant frequency in the population receiving testing, are summarized in Table 3. Overall, the 5,531 high‐confidence variants were detected 2,004,273 times over a one‐year period. For the 13,872 cases with a positive variant according to the ClinVar consensus, 5,083 (36.6%) was also called positive by the automated method. For the 1,990,401 cases with a negative variant according to the ClinVar consensus, 1,989,157 (99.9%) were also called negative by the automated method. Overall, for all 2,004,273 variants, the interpretation of 1,994,240 (99.5%) were concordant between the automated and consensus manual curation process.

**TABLE 3:** Concordance rates between the fully automated and manual high‐confidence variant classifications, on a per‐case basis

	High‐confidence manually interpreted cases			
		Positive (%)	Negative (%)	Total
Fully automated called cases	Positive	5,083 (36.6)	1,244 (0.01)	
	Negative	8,789 (63.4)	1,989,157 (99.9)	
	Total	13,872	1,990,401	**2,004,273**

## 4 DISCUSSION

The widespread adoption of NGS sequencing in clinical laboratories has led to increased demand for variant interpretation, which has promoted the continued development of automated software to increase the efficiency of variant interpretation (Cox et al., 2016). One study has shown that automated variant curation software resulted in an increase in variant interpretation efficiency by 80% (Cox et al., 2016). However, for platforms that provide a final variant interpretation as part of their workflow, there is little peer‐reviewed evidence assessing automated variant interpretations in a clinical setting (Chunn et al., 2020; Furness, 2016; Perakis et al., 2020; Ravichandran et al., 2019). In this study, we analyzed one year of retrospective clinical data to assess the performance of suggested automated interpretations computed by a commercially available variant interpretation support tool.

Our analysis was based on a subset of 116,721 variants interpreted by both manual and automated processes. These were filtered to consider only those with independent, high‐confidence evidence for interpretation. Of the 5,548 high‐confidence ClinVar‐concordant variants included in our analysis, 22.7% of variants that were manually classified and reported to our patients as positive were computed as negative by the automated process. Of the variants that were manually classified as negative, 1.7% were computed as positive by the automated method (Table 1).

The most frequent cause of discrepancy (46.5%) involved the automated software not invoking clinical case criteria considered applicable by manual review (Table 2). When interpreting variants following the ACMG/AMP guidelines, the final interpretation is determined by invoking criteria based on the algorithm defined by Richards et al. (2015). Briefly, there are 27 criteria, each of which is defined by the strength of evidence (strong, moderate, and supporting) and whether the evidence supports the variant being pathogenic or benign. One of the most important types of criteria in determining the interpretation of the variant is clinical cases, which are generally found in peer‐reviewed publications. To review clinical cases, the automated software must both correctly identify all literature referencing the variant of interest and identify any clinical cases reported within that literature. The automated software identified all relevant peer‐reviewed publications in 166 out of 167 variants, but manual review was required to identify all relevant clinical cases within these publications, which resulted in one or more ACMG/AMP pathogenic case criteria to be invoked in the manual review but that was missed in the automated review. Overlooking clinical cases in the literature resulted in the automated interpretation not invoking clinical ACMG/AMP criteria as defined by Richards et al. (2015), such as those criteria invoked by affected compound heterozygote patients (PM3, moderate level pathogenic evidence as defined in Richards et al., 2015), co‐segregation with disease in multiple affected family members (PP1, supporting level pathogenic evidence as defined in Richards et al., 2015), and unaffected homozygotes (BS2, strong benign evidence as defined in Richards et al., 2015), among others. The most impactful improvement that could be made to automated software is the continued improvement in the analysis of primary literature. It is important to note that automation offers significant benefits in literature retrieval and efficiency of variant interpretation. In a white paper reviewing 2324 variants by an independent clinical laboratory, QCI was shown to identify 13,938 additional article‐variant pairs when compared to another laboratory's manual literature retrieval process (Cox et al., 2016). The second most frequent cause (15.9%) was classifying high‐frequency variants with pathogenic evidence as negative, while manual review considered evidence sufficient to support pathogenicity. In situations like this, the elevated allele frequency in a sub‐population within gnomAD invokes ACMG/AMP criteria for when allele frequency is greater than expected for the disorder (BS1). However, it is unclear if the phenotype prevalence used to determine if BS1 is applicable is from that same sub‐population or from a global population. Strong evidence that the variant is benign pushes the interpretation of the variant to the VUS category in the algorithm. Therefore, high‐frequency pathogenic variants need increased scrutiny. Improvements to automation could be made to consider the global population allele frequency and global phenotype prevalence and to evaluate if the frequency of the variant is too common. Based on our understanding, the automated software uses sub‐populations to evaluate if the allele frequency is too high to represent the global phenotype frequency. Automation has yet to accomplish this level of critical review. The third most frequent cause for discordancy also involved the automated software not invoking functional criteria (e.g., PS3 and BS3) from the literature considered applicable by manual review (10.3%) (Table 2). While additional improvement could be made by updating external databases, such as UniProt and ClinVar, we see this as a minor source of discordance representing only 5.8% of discordances in this study (Table 2) and outside of the control of the automated software developers. Two of the three most common causes of discrepancy were related to the automated software not invoking ACMG/AMP criteria from the literature considered applicable by manual review, which supports the difficulty of automated software reviewing scientific literature. Improvements to automated interpretation of literature would be required to increase the accuracy of automation in the context of variant interpretation.

We found that many higher‐frequency variants were disproportionately represented among variants with discrepant interpretations. Within a clinical population, high‐frequency variants are important because one variant misinterpretation will affect many cases. This explains why 22.7% of the variants determined to be positive by ClinVar manual consensus were classified as negative by the automated method on a per‐variant basis, but this number rose to 63.4% on a per‐case basis (Tables 1 and 3). To have clinical utility in a stand‐alone capacity, we expect automated methodologies would need to demonstrate substantially improved concordance rates with consensus manual variant curation.

The study has some limitations. Primarily, variant curation involves a degree of subjectivity by the professional performing the review, and the true pathogenicity of some variants is not possible to predict with the current information available. However, this subjectivity was minimized by only evaluating variants that had a consensus among at least three labs performing manual curations. There may be some ascertainment bias in the cohort in that manual interpretation teams are likely to put more effort into reviewing variants that are considered positive based on ClinVar, thus uncovering more discrepancies among that cohort. In the variant interpretation process, both the manual and automated processes take ClinVar entries into account and this should result in a high level of concordance. Our data indicate that this is not the case.

We recognize that the automated variant interpretation program used in this study is designed to be a support tool and is not marketed for stand‐alone automation. In fact, the software manual explicitly states that it is “… strongly advised that users review the evidence and use their expert judgment to perform conflict resolution.” Additionally, it is important to note that conflicting criteria in QCI defaults to a negative interpretation, and Qiagen recommends expert review. This suggests Qiagen acknowledges the importance of manual review in variant interpretation, but that may not be the case for other platforms offering automated computed interpretations. Nevertheless, the automated tools employed are analogous to those used in other products marketed to be stand‐alone software. The findings reported here should, therefore, be interpreted as a rationale for, and a method of evaluating, stand‐alone genetic variant interpretation tools prior to clinical implementation.

In summary, we used a high‐confidence dataset of ClinVar variant interpretations as a comparator to assess automated variant interpretation software as a stand‐alone tool. In our retrospective case study analysis, we observed low rates of concordance between automated interpretations and consensus manual interpretations. The concordance rates were considerably lower for variants with positive interpretations according to the consensus manual interpretations. Considering concordance rates on a per‐case basis in a full clinical population further illustrated how the effects of discrepancy in high‐frequency variants can affect many cases. While the automated software functions admirably as a literature retrieval and database mining tool, it struggled to appropriately interpret the information within literature, highlighting the essential nature of manual review of variant data at this time. As automation continues to be integrated into variant curation workflows, and as professional guidelines are revised, external and internal validation of automated outputs will be essential for accurate variant interpretation and reporting.

## AUTHOR CONTRIBUTIONS

Bryan J. Gall, Trevor B. Smart, and Nina Sanapareddy conceptualized the study and Dianne Keen‐Kim, Zachary P. Demko, and Nina Sanapareddy supervised it. Study methodology was designed by Bryan J. Gall, Nina Sanapareddy, Trevor B. Smart, and Robin Munch. Data curation was done by Robin Munch, Supraja Kolluri, Hamsa Tadepally, and Karen Phaik Har Lim, and data analysis was performed by Bryan J. Gall, Trevor B. Smart, Robin Munch, and Nina Sanapareddy. Original manuscript was drafted by Bryan J. Gall and Trevor B. Smart. Bryan J. Gall, Trevor B. Smart, Zachary P. Demko, Peter Benn, Vivienne Souter, and Dianne Keen‐Kim performed manuscript review & editing. All authors reviewed and approved the final version of the manuscript.

## CONFLICT OF INTEREST

All authors (except P.B.) are employed by Natera, Inc., a laboratory that offers fee‐based carrier screening. This employment is noted in the author affiliations. PB is a paid consultant to Natera, Inc. The authors declare no additional conflicts of interest beyond their employment affiliation.

## ETHICS STATEMENT

Data used in this study were de‐identified, and an IRB exemption was obtained for publication of this analysis [E&I Review Services, Corte Madera, CA; IRB number: 17113–05]. Since the data used were de‐identified, the study was deemed not to include human subjects and thus, informed consent was not needed.

## Supporting information


**Table S1** Variants where the Natera reporting differed from ClinVar classification and the reasons for altered reporting

## Data Availability

The data that support the findings of this study are available from the corresponding author upon reasonable request.
