# Supplementary material for: Assessment of an automated approach for variant interpretation in screening for monogenic disorders: A single‐center study
Source: Mol Genet Genomic Med. 2022 Nov 5;10(12):e2085. doi: 10.1002/mgg3.2085 (PMC9747559; doi:10.1002/mgg3.2085)
Supplement: Supplementary file 1 — Table S1 Variants where the Natera reporting differed from ClinVar classification and the reasons for altered reporting [file MGG3-10-e2085-s001.docx]

**Assessment of an automated approach for variant interpretation in screening for monogenic disorders: A single center study**

Bryan J Gall^1^, Trevor Smart^1^, Robin Munch^1^, Supraja Kolluri^1^, Hamsa Tadepally^1^, Karen PH Lim^1^, Zachary P Demko^1^, Peter Benn^2^, Vivienne Souter^1^, Nina Sanapareddy^1^, Dianne Keen-Kim^1^

^1^Natera Inc., Austin, Texas, USA

^2^Genetics and Genome Sciences, University of Connecticut Health Center, Farmington, CT, USA

Corresponding author:

Bryan J. Gall, PhD

Natera, Inc., 13011 McCallen Pass, Austin, Texas, 78753, USA

E-mail: bgall@natera.com

Phone: (510) 826-3572

**Supplementary Information**

**Supplementary Table**

**Table S1.** Variants where the Natera reporting differed from ClinVar classification and the reasons for altered reporting.

| **Manual** | **Automated** | **ClinVar** | **Cause of discrepancy** | **Gene** | **GenBank reference sequence** | **Variant** | **Criteria invoked (manual)** | **Criteria invoked (automated)** |
| --- | --- | --- | --- | --- | --- | --- | --- | --- |
| Positive | Positive | Negative | Multiple clinical cases, but benign functional evidence | *CFTR*  OMIM (602421) | NM_000492.4 | c.3041A>G | PM1, PM2, PM3, PP3, BS3 | PM1, PM2, PM3, PP3 |
| Positive | Positive | Negative | ClinVar submission prior to publication invoking additional criteria | *CFTR*  OMIM (602421) | NM_000492.4 | c.3297C>A | PS3, PM2, PM3, PP3 | PM1, PM2, PM3, PP3 |
| Positive | Positive | Negative | BP2 criteria determined to not outweigh multiple clinical CF cases | *CFTR*  OMIM (602421) | NM_000492.4 | c.473G>A | PM1, PM2, PM3, PP3, BP2 | PM1, PM2, PM3, PP3 |
| Positive | Negative | Negative | ClinVar entries described VOI as VUS - LIKELY PATHOGENIC. Cusp variant | *CFTR*  OMIM (602421) | NM_000492.4 | c.4225G>A | PM1, PM2, PM3, PP3 | PM1, PM2, PP3 |
| Positive | Positive | Negative | Single clinical case, LD decision | *MYO7A*  OMIM (276903) | NM_000260.4 | c.2002C>T | PM1, PM2, PM3, PM5, PP3 | PS4, PM1, PM2, PM3, PM5, PP3 |
| Negative | Negative | Positive | High allele frequency, greater than expected with phenotype frequency. | *RARS2*  OMIM (611524) | NM_020320.5 | c.419T>G | BS1, PM3, PP3 | BS1, PS4, PM3, PP3, PP5 |
| Negative | Negative | Positive | Multiple clinical cases with complex or undefined genotypes | *USH2A*  OMIM (608400) | NM_206933.4 | c.6937G>T | PM2, PP3 | PM1, PM2, PP3 |
| Negative | Negative | Positive | After reviewing manual curation updated to likely pathogenic | *CBS*  OMIM (613381) | NM_000071.3 | c.992C>A | PM2, PM3, PP3, PP5 | PM1, PM2, PP3 |
| Positive | Negative | Negative | After reviewing manual curation updated to VUS | *COL4A3*  OMIM (120070) | NM_000091.5 | c.4665G>A | PM2, BS2, BP6 | PM2, BS2, BP6 |
| Negative | Positive | Positive | Multiple clinical cases with complex or undefined genotypes | *ATP7B*  OMIM (606882) | NM_000053.4 | c.2905C>T | PM2, PM5, PP3 | PM1, PM2, PM5, PP3 |
| Negative | Negative | Positive | ClinVar used in silico data to curate, which was supporting evidence internally | *CEP290*  OMIM (610142) | NM_025114.4 | c.4438-3delC | PM2, PM3, PP3 | PM2, PM3, PP3 |
| Negative | Positive | Positive | Multiple clinical cases with complex or undefined genotypes | *USH2A*  OMIM (608400) | NM_206933.4 | c.14426C>T | PM2, PM3, PP3 | PS4, PM2 PM3, PP3 |
| Positive | Negative | Negative | One single clinical case with functional evidence was identified and determined to be sufficient for interpretation as likely pathogenic | *ATM*  OMIM (607585) | NM_000051.4 | c.8520G>C | PS3, PS4, PM2, PP3 | PM1, PM2, PP3 |
